# Not All Sperm Are Equal: Functional Mitochondria Characterize a Subpopulation of Human Sperm with Better Fertilization Potential

**DOI:** 10.1371/journal.pone.0018112

**Published:** 2011-03-23

**Authors:** Ana Paula Sousa, Alexandra Amaral, Marta Baptista, Renata Tavares, Pedro Caballero Campo, Pedro Caballero Peregrín, Albertina Freitas, Artur Paiva, Teresa Almeida-Santos, João Ramalho-Santos

**Affiliations:** 1 Department of Life Sciences, Center for Neuroscience and Cell Biology, University of Coimbra, Coimbra, Portugal; 2 Human Reproduction Service, University Hospitals of Coimbra, Coimbra, Portugal; 3 Human Reproduction Unit, Tambre Clinic and Tambre Foundation, Madrid, Spain; 4 Histocompatibility Centre of Coimbra, Coimbra, Portugal; 5 Faculty of Medicine, University of Coimbra, Coimbra; McGill University, Canada

## Abstract

Human sperm samples are very heterogeneous and include a low amount of truly functional gametes. Distinct strategies have been developed to characterize and isolate this specific subpopulation. In this study we have used fluorescence microscopy and fluorescence-activated cell sorting to determine if mitochondrial function, as assessed using mitochondrial-sensitive probes, could be employed as a criterion to obtain more functional sperm from a given ejaculate. We first determined that mitochondrial activity correlated with the quality of distinct human samples, from healthy donors to patients with decreased semen quality. Furthermore, using fluorescence-activated cell sorting to separate sperm with active and inactive mitochondria we found that this was also true within samples. Indeed, sperm with active mitochondria defined a more functional subpopulation, which contained more capacitated and acrosome intact cells, sperm with lower chromatin damage, and, crucially, sperm more able to decondense and participate in early development using both chemical induction and injection into mature bovine oocytes. Furthermore, cell sorting using mitochondrial activity produced a more functional sperm subpopulation than classic swim-up, both in terms of improvement in a variety of functional sperm parameters and in statistical significance. In conclusion, whatever the true biological role of sperm mitochondria in fertilization, mitochondrial activity is a clear hallmark of human sperm functionality.

## Introduction

Although all sperm may look the same to a casual observer in fact human ejaculates are very heterogeneous, and subpopulations of sperm with distinct biochemical and physiological characteristics can be identified in every sample. It is actually believed that only a very small percentage of sperm is able to achieve fertilization [1], [2]. In fact, one of the major challenges in modern Andrology is to better characterize and isolate this particular subpopulation.

The issue of why so many male gametes seem non-functional, or what possible advantages might be relevant for a seemingly wasteful process, have led to several proposals, many related to the concepts of “sperm competition” and “sperm selection”. For example, in terms of sperm competition excess numbers of male gametes might favor males in cases of multiple matings, and it has even been suggested that the role of certain subpopulations within the ejaculate could be to disable/destroy sperm from other individuals. On the other hand, sperm selection suggests a role for the female reproductive tract in selecting the best sperm from a large population [for a general overview on these topics see 3]. The need for surplus sperm with distinct motilities to help penetrate the cervical mucus [4], and the concept of activating distinct subpopulations at different times [5] might also be envisioned, so that passage to the oviduct is guaranteed for some cells, and that viable sperm would be present for longer periods after mating in case ovulation did not occur with the same timing. A related hypothesis, first developed in rabbits, is that most sperm would engage female immunological responses, allowing a minority of less immunoreactive sperm to advance in the reproductive tract [6]. Indeed, when collected from the oviduct of an inseminated female a minor sperm population was able to successfully navigate a second reproductive tract and was more likely to fertilize oocytes than sperm from a non-selected ejaculate [7]. Since these pioneering experiments much work has been carried out to determine the exact characteristics of this putative sperm subpopulation.

Importantly, high throughput “omics” analysis of sperm aimed at determining what makes the male gamete functional (either for clinical purposes, or using sperm as a simple cellular model with a limited array of functional characteristics) rely on bulk samples when, as noted, only a subset of the cells analyzed are actually functional, thus possibly resulting in a skewed analysis. Distinct techniques can be used to fractionate human sperm. Among them density gradient centrifugation (DGC) and swim-up (as a stand-alone, or after DGC) are the standard preparation techniques, routinely used to select sperm for assisted reproduction technologies (ART). The first enables, not only to separate sperm from both seminal plasma and round cells [8], but also to isolate a fraction of mature, motile, morphologically normal sperm [9,reviewed in 10]. Indeed the higher density layer seems to be enriched in viable sperm when compared to neat semen or to the lower density layer [11], [12], and also to be more responsive to induced capacitation, a process sperm needs to undergo (*in vivo*, in the female reproductive tract, or *in vitro*) before being able to fertilize an oocyte [13], [14], [15]. Additionally, the recovered subpopulation was shown to possess more sperm with functional mitochondria [11], [16], [17], lower levels of reactive oxygen species (ROS) [11] and decreased amounts of sperm with apoptotic and necrotic markers [12], [17]. An increase in chromatin integrity has also been reported [11], [16], [18], [19], although this had been contradicted by others [20], [21]. The swim-up procedure, which is based on the movement of sperm to an overlaying medium [22], is characterized by the recovery of a subpopulation enriched in highly motile and morphologically normal cells [10], [23]. When compared to the subpopulation of sperm that did not migrate the migrated subpopulation was shown to have better kinetic properties [14], higher viability [12], less sperm with apoptotic and necrotic markers [12], [23], [24], and increased levels of the hyperactivation motion critical for fertilization [14]. However no differences were found in the percentage of tyrosine-phosphorylated sperm, the most common marker for capacitation [25]. Similarly to what was described for gradient selection, results related to chromatin integrity seem controversial [18], [21], [26], [27].

Recent developments in sperm biology have led to the development of novel selection strategies for use in ART. For instance, sperm with phosphatidylserine in the outer leaflet of the plasma membrane (an early apoptotic event in other cell types) can be eliminated by using either annexin-V-conjugated microbeads and Magnetic Cell Sorting (MACS) [28] or annexin-V labeling and Fluorescence Activated Cell Sorting (FACS) [29]. Annexin-negative subpopulations, enriched in viable sperm, also seem to have more sperm with normal morphology [29], [30], as well as more motile cells [31], although this last result has been questioned [30], [32]. At any rate, this method allows the recovery of sperm with lower levels of activated caspases [33], lower DNA fragmentation, more functional mitochondria [32], [34] and higher ability to undergo both capacitation and the acrosome reaction (a sperm-specific secretory event crucial for oocyte penetration) [34], [35]. Interestingly, annexin-negative sperm seem to have higher oocyte penetration capacity compared to annexin-positive sperm, as well as higher chromatin decondensation after Intracytoplasmatic Sperm Injection (ICSI), an ART technique used to inject a single sperm into an oocyte when normal fertilization is impaired [36]–[37]. Moreover, when performed before DGC, MACS with annexin V seems to increase cleavage and pregnancy rates after ICSI [30]. In a related application FACS using membrane permeant DNA binding dyes has been widely and successfully used to separate X- and Y-chromosome bearing sperm for animal reproduction purposes [reviewed in 38].

Although the fractionation techniques described above can be used to obtain subpopulations with improvements in certain sperm parameters, when compared to native semen, a subpopulation including only fertile sperm has never been isolated. This is mainly due to the fact that we are still not able to completely describe what makes a competent spermatozoon. Furthermore, in all likelihood such ideal sperm segregation would most probably imply the successive use of a series of procedures.

Would mitochondrial functionality be an adequate criterion to distinguish better quality sperm subpopulations? There are a number of reasons to believe that this may be the case. First, cumulative outcomes strongly suggest that mitochondrial functionality is intimately associated with both sperm quality and fertilization ability [for a review see 39]. Secondly, the heterogeneity underlying any ejaculate is also reflected in differences in mitochondrial-related traits. For instance, we have previously shown that the percentage of sperm expressing certain mitochondrial proteins (including different subunits of cytochrome c oxidase, one of the complexes constituting the electron transfer chain, and DNA polymerase gamma, the sole mitochondrial DNA polymerase) is variable. Any given sample will posses sperm that express and sperm that do not express these proteins, and the proportion of each seems to mirror sample quality [40]. The same is also true for mitochondrial membrane potential (MMP), a more general indicator of mitochondrial functionality, which can be monitored using a variety of cationic and lipophilic fluorescence probes; in that both cells with polarized and cells with non-polarized mitochondria will always co-exist in an ejaculate [41]. Finally, the outcomes of two reports using FACS with different mitochondrial probes suggest that subpopulations of sperm with higher MMP might have an enhanced fertilization potential. The first relied on the segregation of sperm according to rhodamine 123 (Rh123) staining, and hinted that FACS has no detrimental effect on sperm [42]. Moreover, Rh123 positive subpopulations were shown to be enriched in viable and motile gametes. Likewise, others have evidenced that subpopulations of sperm with higher MMP (isolated using either 3,3′-dihexyloxacarbocyanine iodide or chloromethyl-x-rosamine) also have more motile and morphologically normal gametes, with a higher capacity to respond to induced acrosome reaction [43]. Additional analyses are however needed to further support these studies and clearly understand whether sperm with high MMP would constitute subpopulations with truly improved quality, including assays related to sperm functionality in terms of early development.

With this in mind, and using fluorescence microscopy and MMP-disrupters with diverse modes of action, we have previously tested the ability of various mitochondrial probes to monitor human sperm MMP [41]. Here we have selected MitoTracker Green (MT-G) to isolate sperm subpopulations through FACS, since a) this probe stains human sperm mitochondria according to MMP; b) all sperm samples exhibit two MT-G subpopulations: MT-G positive and MT-G negative; c) MT-G non-specific staining (i.e, staining in the sperm tail) is negligible. The two subpopulations obtained (sperm with high MMP and sperm with low MMP) were then compared for a comprehensive battery of parameters known to be required for sperm function. We aimed to determine whether sperm with functional mitochondria would constitute subpopulations with increased fertilization potential.

## Results

### MitoTracker Green FM staining and sperm quality

The assessment of mitochondrial membrane potential (MMP) is easily carried out using specific cationic probes such as carbocyanine, rhodamine and rosamine derivatives, and we have previously shown that several of these probes, including MitoTracker Green (MT-G), accurately monitor the MMP of a sperm sample at the time of incubation, staining the midpiece of a variable proportion of sperm [41].

**Figure 1:**
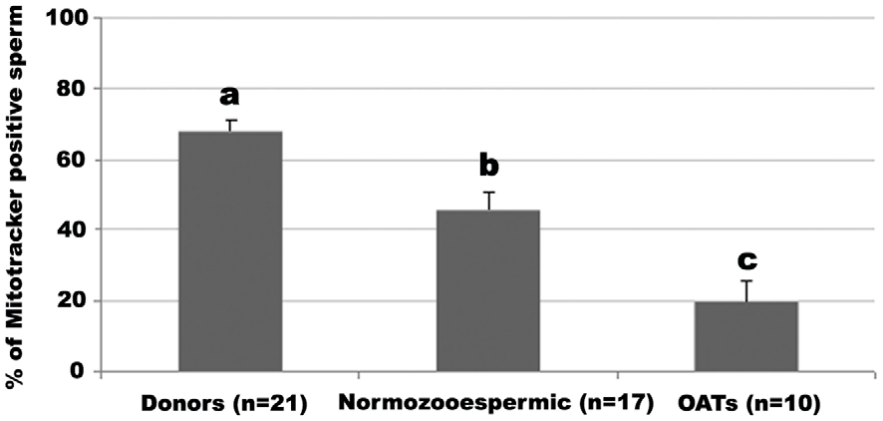
Comparison between different quality samples for the percentages of sperm stained with MitoTracker Green. Data are expressed as mean ± SEM. n = number of samples used. a ≠ b (*P* = 0.002); a ≠ c (*P*<0.001) b ≠c (*P* = 0.003).

**Figure 2:**
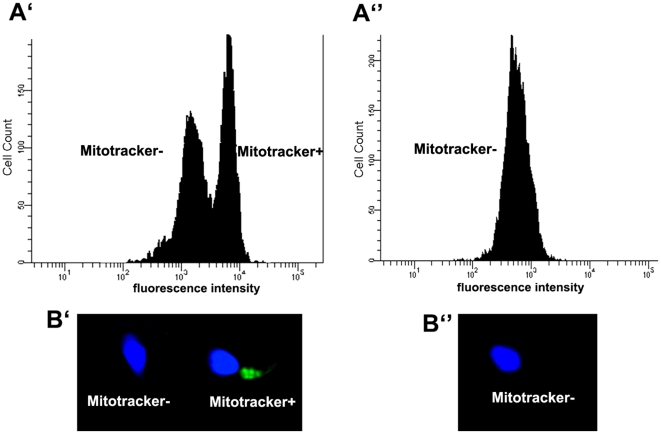
Sperm sample incubated with MitoTracker Green. **A**. Representative histograms obtained using flow cytometry **A′**) Two subpopulations are obtained and can be sorted: MitoTracker positive (stained sperm) and MitoTracker negative (unstained sperm); **A″**) Sperm sample incubated with MitoTracker Green and FCCP, a MMP-disrupter. MitoTracker green labelling patterns observed by fluorescence microscopy: **B′**) MitoTracker positive (stained sperm) and MitoTracker negative (unstained sperm) sperm; **B″**) MitoTracker negative (unstained) sperm observed after incubation with MitoTracker Green and FCCP. For imaging purposes, nuclear DNA was stained with the DNA dye Hoechst 33342 (Molecular Probes).

In the present study, and in order to establish whether MT-G staining is associated with sperm quality, we have used fluorescence microscopy to compare three groups of freeze-thawed samples: healthy sperm donors regularly used in artificial insemination procedures, and two infertility patient groups, normozoospermic patients and oligoasthenoteratozoospermic (OAT) patients. The former have no obvious sperm quality issues as determined using World Health Organization (WHO) criteria, while the latter are patients with clear sperm quality issues in terms of a poor rating on all three main parameters routinely monitored: sperm quantity (<15×10^6^ sperm/ml; or <39×10^6^ total sperm number/ejaculate), sperm motility (<32% progressively motile sperm), and sperm morphology (<4% normal forms). As anticipated, the mean percentage of sperm stained with MT-G (assessed by fluorescence microscopy; Fig. 2B′) was higher for the donor group and lower for the OAT samples (*Ps* <0.01; Fig. 1). The distinctiveness of each group was also evident when comparing other well established sperm functional parameters (viability, capacitation status, acrosome integrity and chromatin integrity; data not shown) although differences were more significant for MT-G staining. Comparable results were only obtained for viability: the mean % of sperm with integral membrane was distinct in the three groups (64.6±3.5 for donors, 44.9±5.5 for normozoospermic and 19.5±6.5 for OATs; Ps <0.05). Furthermore, positive correlations were found between MT-G staining and the other sperm functional parameters (Table 1) i.e., samples with higher % of MT-G positive sperm had an increased likelihood of having more viable sperm, with increased motility, enhanced ability to capacitate, and increased chromatin and acrosome integrities These results suggest that the percentage of MT-G stained sperm of a given (bulk) sample reflects its quality. Therefore, MT-G positive sperm may constitute a better quality, more functional, sperm subpopulation.

**Table 1:** Correlations between MitoTracker Green positive sperm and several sperm quality parameters*.

Progressive motility	Intact membrane	COX I expression	Intact acrosome	Capacitated	Chromatin integrity
0.811 (*<0.001*)	0.811 (*<0.001*)	0.616 (*<0.001*)	0.421 (*0.004*)	0.440 (*0.002*)	0.290 (*0.046*)

The percentage of MitoTracker Green positive sperm of a given sample positively correlates with the percentage of sperm with a) progressive motility; b) intact membrane; c) COX I expression; d) intact acrosome; e) phosphotyrosines f) integral chromatin.

*Correlations (*R*; upper numbers) and *P* values (lower numbers).

### Isolation of subpopulations of sperm using MT-G and a cell sorter

To corroborate our hypothesis, we used a cell sorter to fractionate human sperm samples previously incubated with MT-G. By doing so we were able to isolate two distinct subpopulations: MT-G positive sperm and MT-G negative sperm (Fig. 2A′). Noteworthy, viability monitored in both subpopulations was indistinguishable (44.2±3 for MT-G positive and 40.1±3.4 for MT-G negative; p>0.5, n = 10), suggesting that the process is not merely selecting viable cells.

**Figure 3:**
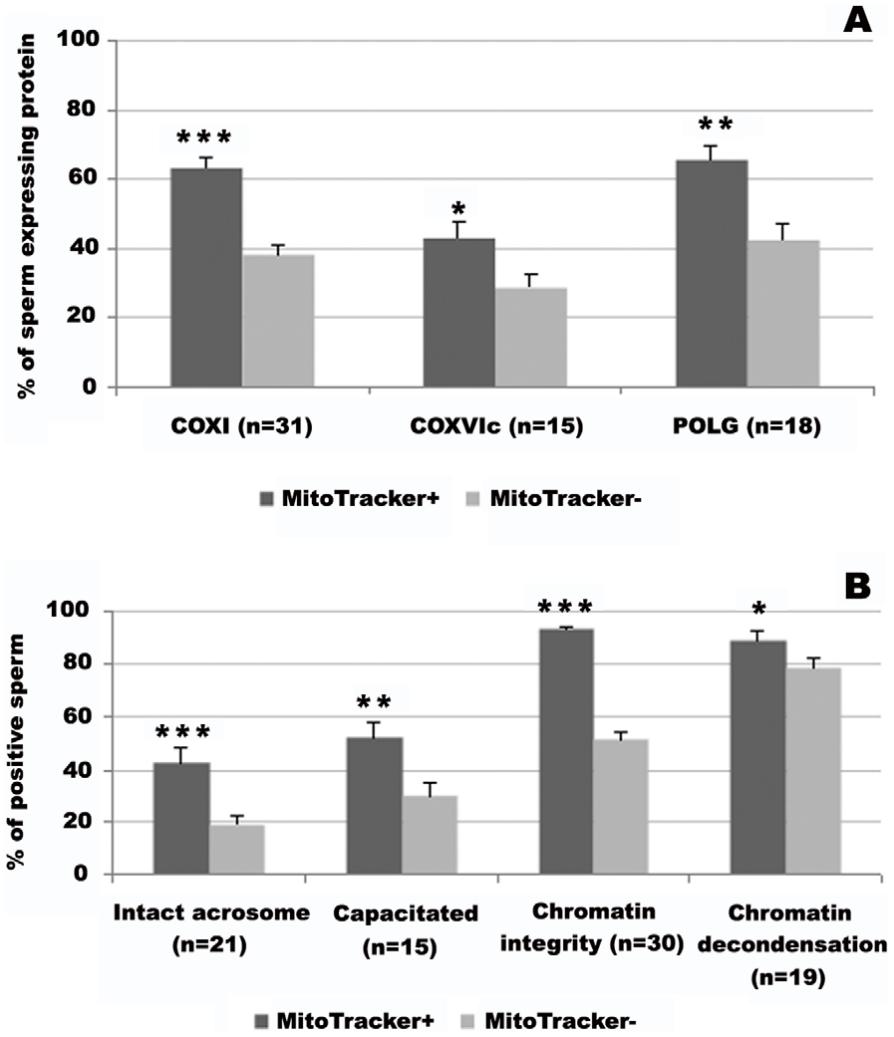
Comparison between subpopulations of MitoTracker positive and MitoTracker negative sperm. **A**. Expression of the mitochondrial proteins COX I, COX VIc and POLG. Data are expressed as mean percentage of sperm ± SEM. **B**. Functional parameters: acrosome integrity, capacitation status, chromatin integrity and chromatin decondensation ability. Data are expressed as mean ± SEM of the percentage of: sperm with intact acrosome; capacitated sperm; sperm with integral chromatin; and sperm responsive to chemically induced chromatin decondensation. n =  number of samples used in each experiment. (*** *P*<0.001; **P<0.01; *P<0.05).

With the aim of confirming that the subpopulations isolated by MT-G FACS have distinct mitochondrial characteristics, we evaluated the expression of three mitochondrial proteins, the presence of which we have previously associated with sperm quality [40]: two subunits of cytochrome c oxidase, namely COX I (encoded by the mitochondrial genome) and COX VIc (nuclear encoded), and DNA polymerase gamma (POLG). As expected, the percentage of sperm expressing each of these proteins, as determined by immunocytochemistry, was significantly higher in MT-G positive compared with MT-G negative sperm (Fig. 3a), confirming that the two isolated subpopulations have distinct mitochondrial attributes.

### Comparison between MT-G positive and MT-G negative sperm subpopulations

#### Capacitation, acrosome integrity and chromatin integrity

To determine if, besides mitochondrial activity, MT-G positive sperm have better characteristics than MT-G negative sperm, we have compared the two subpopulations for various functional parameters, namely capacitation, acrosomal status and chromatin integrity. Uncapacitated acrosome-less sperm will be unable to fertilize, while chromatin damage will likely interfere with embryo developmental. The MT-G positive subpopulation contained significantly more capacitated sperm (mean ± SEM  = 42.8±6.1 *versus* 19.4±3.1; *P*<0.001) more sperm with intact acrosomes (52.5±5.5 *versus* 30.3±4.9; *P* = 0.005) and a much higher proportion of sperm with integral chromatin (93.3±1.0 *versus* 51.8±2.8; *P*<0.0005; Fig. 3b).

#### Chromatin decondensation ability

To determine if one subpopulation of sperm is truly more fertilization-competent than another functional assays are required. To further establish if MT-G positive sperm subpopulations are enriched in good quality sperm chromatin decondensation ability was also monitored. Decondensation is essential for the formation of the male pronucleus and, consequently, for syngamy to take place. Given that the biologically correct assay (*i.e.* fertilization ability assayed with human oocytes) was not possible for obvious ethical reasons, two different approaches were employed.

**Figure 4:**
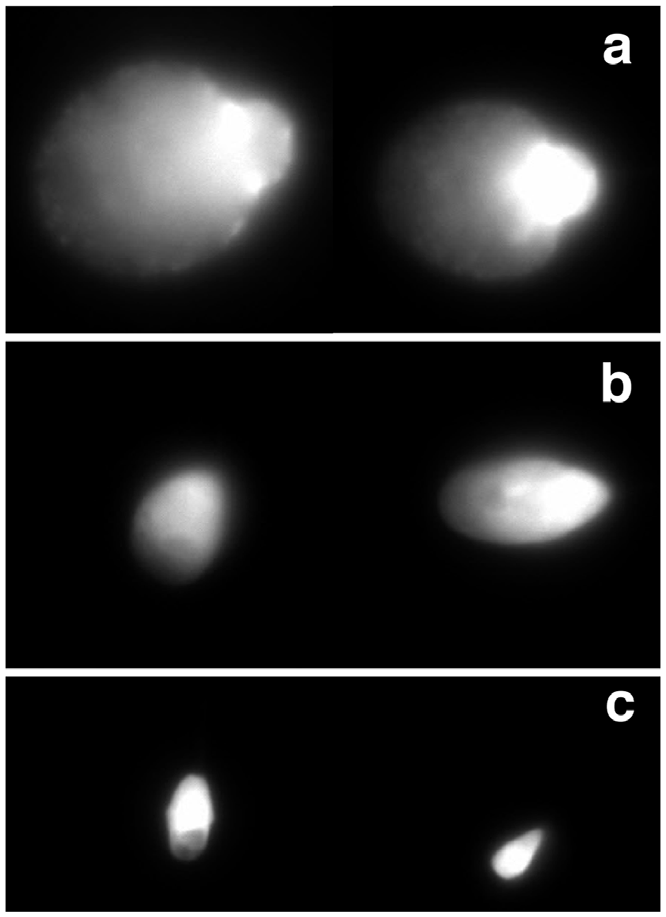
Chemically induced sperm chromatin decondensation (after treatment with Heparin and GSH). Three degrees of decondensation were observed: grade a) very decondensed; grade b) decondensed; grade c) non decondensed.

The first consisted of chemical-induced decondensation, with the incubation of sperm with heparin and reduced glutathione, based on a published protocol [44], [45], but with modifications. Noteworthy, our protocol included a permeabilization step before adding the inducers. By doing so, we were able to exclude the influence of membrane integrity (otherwise, sperm with a more damaged membrane would be more accessible to the inducers). Samples treated in this way presented sperm with different degrees of decondensation (Fig. 4): very decondensed (grade a), decondensed (grade b) and non decondensed (grade c; similar to the controls – absence of inducers). One logical criticism of this protocol is that it might simply mirror chromatin status and amenability to decondensation, which would result, for example, in sperm with fragmented DNA or with errors in DNA packaging decondensing more extensively. To definitely exclude this possibility samples that were not separated by MT-G FACS (n = 4) were pre-incubated with DNase or H_2_O_2_, or subjected to high temperature in order to effectively damage sperm chromatin using different agents, as previously shown [46]. For all three conditions the percentage of sperm with decondensed chromatin was lower than in the controls (data not shown). This suggests that our novel test is accurate in predicting true chromatin decondensation ability, and not mere chromatin damage or accessibility. We then employed this test to compare sperm subpopulations (Fig. 3b), and found that the percentage of sperm able to decondense its chromatin (grades a and b) was higher in MT-G positive subpopulations (89.1±3.3) when compared with MT-G negative subpopulations (78.7±3.8; *P* = 0.045).

In addition to the *in vitro* chemical test, chromatin decondensation ability was also monitored after injecting human sperm into metaphase-II bovine oocytes in a cross-species “minotaur” assay. This assay evolved from other conceptually similar ones, proposed to study human sperm characteristics and function in terms of oocyte activation, and considering the general unavailability of human oocytes. These included the zona-free hamster oocyte penetration test [47] and ICSI into mouse oocytes [48]. The bovine species was chosen given that rodents, while certainly valuable as models, are not fully representative of most mammals (including humans) in what regards to oocyte activation and early development, namely considering centrosomal inheritance [49].

**Figure 5:**
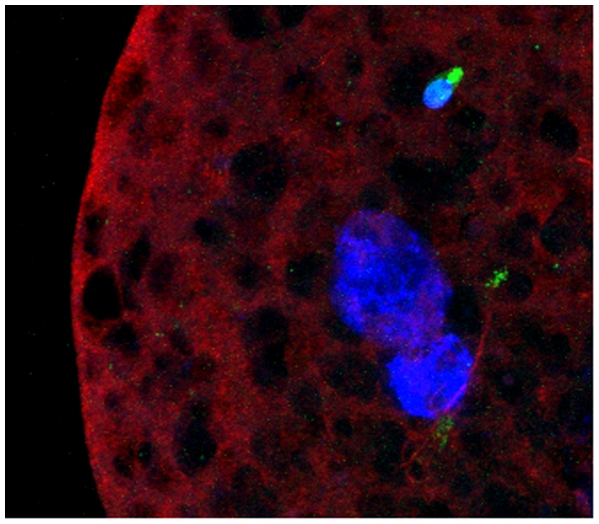
Sperm chromatin decondensation ability monitored after ICSI into metaphase-II bovine oocytes. A non-deconsended sperm (upper) and two decondensed sperm heads (lower) are shown. Nuclear DNA is stained with DAPI (blue); oocyte and sperm tubulin were detected by immunocytochemistry (red) and the sperm midpiece is stained with MitoTracker Green (green).

In our cross-species experiments analysis of bovine oocytes microinjected with human sperm showed a variable extent of chromatin decondensation 24 h after ICSI (Fig. 5). Outstandingly, the percentage of decondensed sperm was significantly higher for MT-G positive sperm (Table 2; *P* = 0.004). Noteworthy, sperm injected into immature non-fertilization competent bovine oocytes (negative control) did not show any type of decondensation (data not shown).

**Table 2:** Sperm chromatin decondensation after ICSI using bovine oocytes.

	Decondensed sperm	Non-decondensedsperm
MitoTracker + (n = 96)	69 (71.9%)	27 (28.1%)
MitoTracker – (n = 100)	52 (52.0%)	48 (48.0%)

Status of human sperm injected into metaphase-II bovine oocytes 24 h after ICSI: n =  number of injected sperm. upper values – number of sperm; lower values – percentages of sperm.

### MT-G FACS *versus* conventional Swim-up

**Figure 6:**
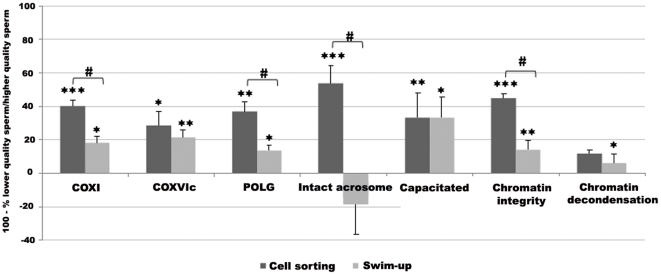
Comparison between sperm fractionation methods: cell sorting after MitoTracker Green staining *versus* swim-up. Bars represent mean ± SEM of 100 – (MT-G negative sperm/MT-G positive sperm x 100) and 100 – (Non migrated sperm/Migrated sperm x 100). Asterisks denote differences between subpopulations: MitoTracker positive compared to MitoTracker negative sperm; migrated compared to non migrated sperm (*** *P*<0.001; **P<0.01; *P<0.05).Cardinals denote differences between the fractionation methods (*P*s <0.001). Sample numbers  = 30 for COX I expression, COX VIc expression and chromatin decondensation, 34 for POLG expression, 15 for acrosome integrity, 20 for capacitation status and 40 for chromatin integrity.

To determine whether MT-G subpopulations obtained by FACS would be enriched in functional sperm when compared to subpopulations obtained using other sperm fractionation techniques, we compared our sperm sorting approach with conventional swim-up. As expected, migrated sperm seems to constitute a better subpopulation than non migrated sperm: there were significant differences for almost all the parameters evaluated, except for acrosome integrity and chemically induced chromatin decondensation ability (Fig. 6). To appreciate how much MT-G positive sperm and migrated sperm constitute better subpopulations when compared to MT-G negative sperm and non migrated sperm, respectively; and also to compare the two fractionation methods, results were expressed as follows: 100 – (MT-G negative sperm/MT-G positive sperm x 100) and 100 – (non migrated sperm/migrated sperm x 100). For most of the parameters analyzed, the improvement in sperm quality was higher for MT-G positive compared to MT-G negative subpopulations than for migrated *versus* non migrated sperm. Indeed, there were significant differences between the two fractionation techniques for the percentage of sperm expressing COX I and POLG, and for the percentage of sperm with an intact acrosome and intact chromatin (*P*<0.001).

Finally, and to determine whether MT-G positive sperm would constitute a better subpopulation than migrated sperm, we compared the chromatin condensation ability of these two subpopulations after ICSI using the “minotaur” assay. For the 57 migrated sperm injected into bovine oocytes, 25 (43.9%) showed decondensation. Such a proportion was significantly lower than the equivalent for MT-G positive sperm (71.9% of sperm decondensed after ICSI; *P* = 0.001).

## Discussion

The intrinsic heterogeneity of an ejaculate is not only reflected by the sex chromosomes (X- and Y-bearing sperm), but by a multitude of sperm quality parameters, which are extremely variable. For instance, motile and immotile sperm, morphologically normal and abnormal sperm, live and dead sperm, sperm with or without apoptotic markers, and so forth, invariably co-exist in a same sample. Certainly, the subpopulation of functional sperm (*i.e.*, sperm that is able to fertilize) constitutes a small proportion of the whole population [1], [2], and distinct criteria can theoretically be used to segregate this subpopulation. However none of them seems to be absolute.

In the current study we sought to determine if mitochondrial membrane potential (MMP), and thus mitochondrial functionality [39], could be used as a criterion to efficiently fractionate and characterize more functional human sperm. We showed that, although the percentage of sperm with active mitochondria was variable from sample to sample, mitochondrial function seemed to mirror sperm quality, with better quality samples having a higher percentage of stained cells. Furthermore, significantly positive correlations were observed between sperm MMP and other parameters known to be required for sperm functionality (motility, viability, capacitation status, acrosome and chromatin integrity). These results strongly suggest that mitochondrial status reflects the state of other fundamental attributes, and is thus a good candidate as a criterion to segregate more functional sperm subpopulations.

Indeed, FACS of sperm pre-incubated with MT-G constitutes an easy and efficient way of isolating sperm subpopulations. While others have separated sperm samples stained with other mitochondrial probes [42], [43], functional assays related to fertilization ability were never performed. Therefore, we compared the two different subpopulations obtained with our approach for a number of sperm facets, and our data clearly shows that sperm with active mitochondria are of superior quality.

The expression of specific mitochondrial proteins associated with sperm quality (COX I, COX VIc and POLG) [40] showed that MT-G positive sperm had more cells expressing each of the three proteins, thus suggesting that the expression of these proteins might contribute for the maintenance of a high MMP in human sperm. Importantly, MT-G positive subpopulations also had a higher percentage of capacitated sperm, sperm with intact acrosomes and intact chromatin, which are known pre-requisites for both fertilization and embryo development. The results for chromatin integrity were particularly informative, suggesting that sperm with functional mitochondria also have integral chromatin, and stressing a relationship between these two parameters.

Even more significant, MT-G positive sperm was shown to be more responsive to chromatin decondensation stimuli, suggesting that this subpopulation would be more competent to form a male pronucleus, and consequently to perform syngamy. Noteworthy, the data obtained *in vitro* (chemical decondensation) were confirmed *in vivo* (ooplasm- induced decondensation). Our novel *in vitro* test constitutes a simple, rapid and cheap functional assay to evaluate sperm chromatin decondensation ability. The *in vivo* approach, while more laborious, resulted in more significant outcomes, clearly showing that sperm with functional mitochondria are more likely to undergo chromatin decondensation and thus participate in early development. Analogous results obtained comparing subpopulations sorted by Annexin-V MACS were controversial [36], [37], suggesting that fractionation of sperm according to mitochondrial activity leads to more homogeneous subpopulations.

The comparison between MT-G FACS and the classical swim-up sperm separation method also stressed the power of the first in attaining sperm with more uniform characteristics. Unsurprisingly, sperm fractionated accordingly to the ability to swim resulted on the isolation of two different quality subpopulations, with migrated fractions showing better features compared to non migrated for almost all parameters analyzed, which is in accordance with published data [12], [14], [21], [23]. However, the distinction between MT-G positive and MT-G negative sperm was much more pronounced, both in terms of parameters monitored and statistical significance, than the one observed by comparing migrated and non migrated sperm. More importantly, MT-G positive sperm seems to be more likely to form a male pronucleus than migrated sperm.

Our data validates the evaluation of MMP as a consistent indicator of sperm quality. It therefore suggests that MMPcan be used as a tool to isolate the best sperm in a sample, given that sperm with functional mitochondria seem to also have other important cellular attributes, and to constitute a more functional subpopulation, most likely with a higher fertilization potential. Others have shown that both sperm quality and fertilization ability (measured as fertilization rates after *in vitro* fertilization - IVF) are strongly related with MMP [11], [50], [51], and, although this was not the goal of the current study, it is thus tempting to indicate mitochondrial probes as possible tools to select the best sperm for ART. The detrimental effects of the use of sperm stained with these probes in ART are theoretically low, as it is well established that sperm mitochondria enter the oocyte upon fertilization but are degraded shortly thereafter [52]. Regardless, appropriate controls would need to be performed. Additionally, and in order to narrow-down the specific subpopulation of sperm with fertilization competence within an ejaculate further fractionation of MT-G positive sperm using other theoretically non-harmful sperm functional markers should be considered.

It is important to note that further studies are also required to define what the precise roles of mitochondria in sperm function are. In fact, and although mitochondria are very efficient on the production of ATP by oxidative phosphorylation (OXPHOS), the nature of the ATP that supports distinct sperm attributes, including motility, is not consensual [for a review see 39]. At any rate, whatever their biological function, active mitochondria characterize fully functional male gametes. Furthermore, efforts in terms of dissecting the characteristics of functional sperm at the cellular, biochemical, metabolic, epigenomic and molecular levels using any variety of “omics” approach (either for ART purposes or using sperm as a simple cellular model) should focus on better quality subsets of an ejaculate, rather than relying on bulk samples, which inevitably include a very high percentage of non-functional male gametes, thus confounding the analysis. The same will be true for other similar mammalian samples. Indeed, the lack of predictive fertility value of many proposed sperm quality parameters may be due to the fact that a mixed population of functional and non-functional spermatozoa is almost always considered.

## Materials and Methods

### Chemicals

All chemicals were supplied by Sigma-Aldrich (St. Louis, MO, USA), unless stated otherwise.

### Biological Material

Fresh semen samples were used in almost all the experiments performed. The first set of data (Results section “MitoTracker Green FM staining and sperm quality”) relied, however, on the use of freeze-thawed samples, as fertile donors were included in this part of the work and only frozen samples were available.

#### Fresh samples

Patients were recruited from the Fertility Clinic (University Hospitals of Coimbra, Portugal) and were undergoing routine semen analysis or fertility treatment involving either *in vitro* fertilization (IVF) or intracytoplasmic sperm injection (ICSI). Informed consent was obtained from all patients, who signed informed consent forms for this purpose, approved by the Institutional Review Board (IRB) of the University Hospitals of Coimbra. All human material was used in accordance with the appropriate ethical guidelines provided by the IRB of the University Hospitals of Coimbra, who also approved the study. Fresh semen samples were obtained by masturbation after 3 to 5 days of sexual abstinence and routine seminal analysis was performed according to the World Health Organization Guidelines [53]. Semen samples were prepared by density gradient centrifugation as described elsewhere [40] and sperm were then washed and incubated at least for three hours in Sperm Preparation medium (SPM; Medicult, Jyllinge, Denmark), which induces capacitation.

#### Freeze-thawed samples

For the initial experiments semen samples were also obtained from consenting healthy donors involved in Spanish sperm donation programs and patients undergoing fertility treatment in another Fertility Clinic (Tambre Clinic, Madrid, Spain). Informed consent was obtained from all patients, who signed informed consent forms for this purpose, approved by the Institutional Review Board (IRB) of the Tambre Clinic. All human material was used in accordance with the appropriate ethical guidelines provided by the IRB, who also approved the study. Routine seminal analysis was performed according to the WHO [53] recommendations. Samples were frozen in TEST buffer with 6% (w/v) glycerol and 10% (w/v) egg yolk as cryoprotectants, as previously described [54], and were kept in liquid nitrogen until further processing. Samples were then thawed by incubation in a water bath for 10 min at 37°C, and prepared by density gradient centrifugation. Sperm were then washed and incubated at least for three hours in SPM.

### MitoTracker Green staining

Live sperm suspensions (10 millions of sperm/mL) were incubated with 20 nM MitoTracker Green FM (Molecular Probes, Eugene, Oregon, USA) for 20 min at 37°C, as previously described, including all the appropriate controls using specific mitochondrial inhibitors to ensure staining was dependent on mitochondrial activity [41]. Samples were either analyzed by fluorescence microscopy (to determine the percentage of MT-G positive sperm – first set of experiments) or subsequently isolated using FACS (see below). In the first case, samples were examined using a Zeiss Axiophot II microscope (Carl Zeiss, Göttingen, Germany) equipped with a triple band pass filter, and the percentage of stained sperm was determined by counting 200 sperm per coverslip, in at least four different fields. In all similar experiments (noted below) counts were performed by at least two observers, and samples were blinded. Negative control experiment was performed by pre-incubating sperm with the MMP-disrupter FCCP (1 µM, 2 h), as previously described [41] (Fig. 2A″ and 2B″).

### Isolation of sperm subpopulations

#### With distinct mitochondrial membrane potential

To separate two subpopulations of sperm with distinct mitochondrial membrane potential, live sperm suspensions were stained with MitoTracker Green, as described above, and were then sorted with a BD FACSAriaTM cell-sorting system (BD Biosciences, Becton, Dickinson and Company, NJ, USA) at a 488 nm wavelength and with the followings settings: laser power - 13 mW; nozzle - 70 µm; sort setup - medium; sheath pressure - 34.50; frequency - 60.0; flow rate: - 1-3 (maximum of 7000 events/sec); and precision - 0160.

#### With distinct progressive motility (Swim-up)

To separate subpopulations of sperm with distinct progressive motility, samples were processed by the routine swim-up procedure using Sperm Preparation medium. Briefly, sperm pellets were gently covered with the medium and incubated for 30 min at 37°C, allowing the motile sperm to swim-up to the medium. For each sample, two subpopulations were isolated: sperm that migrated to the medium (referred here as migrated sperm); sperm that did not migrate (referred herein as non-migrated sperm).

### Sperm quality parameters

#### Membrane integrity

Membrane integrity of either non-fractionated or fractionated sperm (depending on the experiment) was evaluated by fluorescence microscopy using the LIVE/DEAD Sperm Viability kit from Molecular Probes [41]. Detergent permeabilization was used as a positive control. Results were expressed as percentage of viable sperm (i.e, with integral membrane).

#### Expression of mitochondrial proteins

The expression of the mitochondrial proteins cytochrome c oxidase I (COX I) and VIc (COX VIc), and DNA polymerase gamma (POLG) was detected separately by immunocytochemistry using appropriate antibodies (mouse anti-human COX I monoclonal antibody, mouse anti-human COX VIc monoclonal antibody - Molecular Probes - and rabbit anti-human POLG polyclonal antibody - Abcam, Cambridge, UK), as described previously, including all the relevant (secondary antibody-only) negative controls [40]. These analyses were performed in both fractionated and non-fractionated sperm, depending on the experiment.

#### Capacitation and acrosomal status

After incubation in SPM medium for at least three hours, capacitation status was monitored by the detection of phosphotyrosines using rabbit anti-human phosphotyrosine polyclonal antibody (Zymed, South San Francisco, CA, USA). Secondary antibody-only experiments were carried out as negative controls. Acrosomal status was determined separately, using the acrosome content marker *Pisum sativum* agglutinin, linked to fluorescein isothiocyanate (FITC-PSA). Procedures were performed as described before [55] and percentages of capacitated sperm/sperm with intact acrosome were determined. Again, these parameters were analyzed in either non-fractionated or fractionated sperm, as described in the Results section.

#### Chromatin integrity

DNA fragmentation of the distinct sperm subpopulations was monitored using the APO-BrdU TUNEL assay kit (Molecular Probes), as previously described, including negative (lack of one enzymatic component) and positive (artificial induction of DNA fragmentation) controls [56]. Results were expressed as percentage of sperm with intact (i.e.: not fragmented) DNA.

Chromatin status of distinct quality sperm samples was also evaluated using the Diff-Quik stain set (Dade Behring Inc., Newark, USA), as recently established, and using several types of positive controls [46]. This protocol is based on the intensity of nuclei staining: normal sperm nuclei stain lightly, while abnormal nuclei (containing damaged chromatin, *i.e.*, either decondensed or presenting fragmented DNA) stain dark. The percentage of sperm with light nuclei was determined by counting 200 sperm, in at least four different fields.

#### Chromatin decondensation

To evaluate sperm chromatin decondensation ability in the distinct sperm subpopulations, a novel chemical induction *in vitro* test was established, based on published protocols [44], [45]. Briefly, sperm suspensions (10 millions of sperm/mL) previously permeabilized for 20 min with 1% (v/v) Triton X-100 in phosphate buffered saline (PBS, pH 7.2), were incubated with 46 µM heparin (Biochrom AG, Berlin, Germany) and 10 mM reduced glutathione (GSH) for 1 h at room temperature. After stopping reaction with a 5 min incubation with 2% formaldehyde in PBS, samples were stained with the nucleic acid-stain 4,6 diamidino-2-phenylindole (DAPI; Vector Laboratories, Burlingame, CA, USA) and observed using fluorescence microscopy by two independent observers. Results were expressed as percentage of sperm with decondensed chromatin after counting 200 sperm. Each experiment was done in duplicate, and negative controls (incubation in PBS without heparin and GSH) were performed in every single experiment. In order to validate the test (see Results), before permeabilization, 4 samples were pre-incubated: a) with 50 U/mL DNase I for 10 min (to induce DNA fragmentation); b) with 0.5% (v/v) H_2_O_2_ overnight (induction of apoptosis-like damage); c) 5 min at 75°C (to induce temperature-dependent chromatin decondensation).

For *in vivo* sperm decondensation the “Minotaur Assay” was developed by introducing human sperm into bovine oocytes by intracytoplasmic sperm injection (ICSI). Bovine ovaries were obtained at Mapicentro, SA (Leiria, Portugal), a local slaughterhouse. The ovaries were discarded/non-commercial material, non-proliferating oocytes were isolated from them and no cell lines were generated (see below). Bovine ovaries were collected from animals cleared for use by the Portuguese National Veterinary Board and sacrificed according to their guidelines. The use of bovine material followed the ethical and safety guidelines mandated by the Center for Neuroscience and Cell Biology, the Foundation of Science and Technology and the National Veterinary Board, and were according to the recommendations of FELASA (Federation of Laboratory Animal Science Associations). The experiments were directly supervised by a researcher (J. R.-S.) with FELASA approval, and certified by the National Veterinary Board for the use of such material.

Bovine ovaries were transported to the laboratory in PBS with 0.15% (w/v) bovine serum albumin (BSA), 0.05 mg/mL kanamycin and 1% (v/v) GIBCO penicillin/streptomycin (Invitrogen, Paisley, UK), within 2 hours. Follicular contents were aspirated from 2–6 mm follicles with a syringe and a 20-gauge needle and transferred to GIBCO HEPES-buffered TCM-199 medium with L-glutamine (Invitrogen) with 0.5% (w/v) BSA and 1% GIBCO penicillin/streptomycin at 39°C. Cumulus-oocytes complexes were matured for 20–24 h at 39°C, 5% CO_2_ in air, in TCM-199 supplemented with 0.25 mM pyruvate, 2% (v/v) penicillin/streptomycin, 10% (v/v) fetal calf serum, 10 µg/mL luteinizing hormone (LH), 2.5 µg/mL follicle-stimulating hormone (FSH) and 1 µg/ml estradiol. Cumulus cells were removed by a brief incubation in SynVitro Hyadase (Medicult) and washed in Universal IVF Medium (Medicult). Second meiotic metaphase arrested oocytes (with a visible polar body) were used for ICSI with the distinct human sperm subpopulations. Each oocyte was injected with 1-5 sperm using a Leica AM 6000 inverted microscope (Leica Mycrosystems, Wetzlar, Germany) equipped with two micromanipulators (Eppendorf AG, Hamburg, Germany) and two microinjectors (Cell Tram Air and Cell Tram Oil; Eppendorf), as described elsewhere [57]. After injection, oocytes were cultured for 24 h in Universal IVF Medium at 39°C, 5% CO_2_. To evaluate sperm chromatin decondensation, oocyte immunocytochemistry using anti-tubulin antibody was performed as described before [58]. Oocytes were observed under a fluorescence microscope and the chromatin of each injected sperm was classified as decondensed or not decondensed. Negative controls (see Results) consisted of sperm injected in immature oocytes (which are unable to decondense sperm chromatin) [59]. All unused ovarian material was immediately discarded following guidelines provided by the Center for Neuroscience and Cell Biology, the National Veterinary Board and the University Hospitals of Coimbra.

### Statistical analysis

Statistical analysis was carried out using SPSS for Windows (version 13, Chicago, IL, USA). All variables were checked for normal distribution using the one-sample Kolmogorov-Smirnov test. One-way ANOVA was used to compare MitoTracker staining in the three groups of different quality samples and post-hoc analyses were done using Tukey's test. Correlations between MitoTracker staining and the various sperm functional parameters were determined by Pearson's test. Independent samples T-test was used to compare the different sperm subpopulations (MitoTracker positive *versus* MitoTracker negative and Migrated *versus* Non Migrated) for all numerical variables. Pearson Chi-square was used to compare the results of sperm chromatin decondensation after ICSI. *P*<0.05 was considered significant.

## References

[1] Holt WV, Van Look KJ (2004). Concepts in sperm heterogeneity, sperm selection and sperm competition as biological foundations for laboratory tests of semen quality.. Reproduction.

[2] Holt WV (2005). Is quality assurance in semen analysis still really necessary? A spermatologist's viewpoint.. Hum Reprod.

[3] Birkhead TR (2000). Promiscuity: An Evolutionary History of Sperm Competition and Sexual Conflict: Faber and Faber..

[4] Katz DF, Brofeldt BT, Overstreet JW, Hanson FW (1982). Alteration of cervical mucus by vanguard human spermatozoa.. J Reprod Fertil.

[5] Austin CR (1965). Fertilisation.. Foundations of Developmental Biology Series.

[6] Cohen J, Tyler KR (1980). Sperm populations in the female genital tract of the rabbit.. J Reprod Fertil.

[7] Cohen J, McNaughton DC (1974). Spermatozoa: the probable selection of a small population by the genital tract of the female rabbit.. J Reprod Fertil.

[8] Gorus FK, Pipeleers DG (1981). A rapid method for the fractionation of human spermatozoa according to their progressive motility.. Fertil Steril.

[9] Pousette A, Akerlof E, Rosenborg L, Fredricsson B (1986). Increase in progressive motility and improved morphology of human spermatozoa following their migration through Percoll gradients.. Int J Androl.

[10] Henkel RR, Schill WB (2003). Sperm preparation for ART.. Reprod Biol Endocrinol.

[11] Marchetti C, Obert G, Deffosez A, Formstecher P, Marchetti P (2002). Study of mitochondrial membrane potential, reactive oxygen species, DNA fragmentation and cell viability by flow cytometry in human sperm.. Hum Reprod.

[12] Ricci G, Perticarari S, Boscolo R, Montico M, Guaschino S (2009). Semen preparation methods and sperm apoptosis: swim-up versus gradient-density centrifugation technique.. Fertil Steril.

[13] Buffone MG, Doncel GF, Marin Briggiler CI, Vazquez-Levin MH, Calamera JC (2004). Human sperm subpopulations: relationship between functional quality and protein tyrosine phosphorylation.. Hum Reprod.

[14] Chantler E, Abraham-Peskir J, Roberts C (2004). Consistent presence of two normally distributed sperm subpopulations within normozoospermic human semen: a kinematic study.. Int J Androl.

[15] Buffone MG, Verstraeten SV, Calamera JC, Doncel GF (2009). High cholesterol content and decreased membrane fluidity in human spermatozoa are associated with protein tyrosine phosphorylation and functional deficiencies.. J Androl.

[16] Donnelly ET, O'Connell M, McClure N, Lewis SE (2000). Differences in nuclear DNA fragmentation and mitochondrial integrity of semen and prepared human spermatozoa.. Hum Reprod.

[17] Barroso G, Taylor S, Morshedi M, Manzur F, Gavino F (2006). Mitochondrial membrane potential integrity and plasma membrane translocation of phosphatidylserine as early apoptotic markers: a comparison of two different sperm subpopulations.. Fertil Steril.

[18] Sakkas D, Manicardi GC, Tomlinson M, Mandrioli M, Bizzaro D (2000). The use of two density gradient centrifugation techniques and the swim-up method to separate spermatozoa with chromatin and nuclear DNA anomalies.. Hum Reprod.

[19] Tomlinson MJ, Moffatt O, Manicardi GC, Bizzaro D, Afnan M (2001). Interrelationships between seminal parameters and sperm nuclear DNA damage before and after density gradient centrifugation: implications for assisted conception.. Hum Reprod.

[20] Zini A, Mak V, Phang D, Jarvi K (1999). Potential adverse effect of semen processing on human sperm deoxyribonucleic acid integrity.. Fertil Steril.

[21] Zini A, Finelli A, Phang D, Jarvi K (2000). Influence of semen processing technique on human sperm DNA integrity.. Urology.

[22] Lopata A, Patullo MJ, Chang A, James B (1976). A method for collecting motile spermatozoa from human semen.. Fertil Steril.

[23] Piomboni P, Bruni E, Capitani S, Gambera L, Moretti E (2006). Ultrastructural and DNA fragmentation analyses in swim-up selected human sperm.. Arch Androl.

[24] Kotwicka M, Filipiak K, Jedrzejczak P, Warchol JB (2008). Caspase-3 activation and phosphatidylserine membrane translocation in human spermatozoa: is there a relationship?. Reprod Biomed Online.

[25] Turner RM, Eriksson RL, Gerton GL, Moss SB (1999). Relationship between sperm motility and the processing and tyrosine phosphorylation of two human sperm fibrous sheath proteins, pro-hAKAP82 and hAKAP82.. Mol Hum Reprod.

[26] Spano M, Cordelli E, Leter G, Lombardo F, Lenzi A (1999). Nuclear chromatin variations in human spermatozoa undergoing swim-up and cryopreservation evaluated by the flow cytometric sperm chromatin structure assay.. Mol Hum Reprod.

[27] Younglai EV, Holt D, Brown P, Jurisicova A, Casper RF (2001). Sperm swim-up techniques and DNA fragmentation.. Hum Reprod.

[28] Grunewald S, Paasch U, Glander HJ (2001). Enrichment of non-apoptotic human spermatozoa after cryopreservation by immunomagnetic cell sorting.. Cell Tissue Bank.

[29] Hoogendijk CF, Kruger TF, Bouic PJ, Henkel RR (2009). A novel approach for the selection of human sperm using annexin V-binding and flow cytometry.. Fertil Steril.

[30] Dirican EK, Ozgun OD, Akarsu S, Akin KO, Ercan O (2008). Clinical outcome of magnetic activated cell sorting of non-apoptotic spermatozoa before density gradient centrifugation for assisted reproduction.. J Assist Reprod Genet.

[31] Said TM, Grunewald S, Paasch U, Glander HJ, Baumann T (2005). Advantage of combining magnetic cell separation with sperm preparation techniques.. Reprod Biomed Online.

[32] de Vantery Arrighi C, Lucas H, Chardonnens D, de Agostini A (2009). Removal of spermatozoa with externalized phosphatidylserine from sperm preparation in human assisted medical procreation: effects on viability, motility and mitochondrial membrane potential.. Reprod Biol Endocrinol.

[33] Paasch U, Sharma RK, Gupta AK, Grunewald S, Mascha EJ (2004). Cryopreservation and thawing is associated with varying extent of activation of apoptotic machinery in subsets of ejaculated human spermatozoa.. Biol Reprod.

[34] Grunewald S, Paasch U, Said TM, Rasch M, Agarwal A (2006). Magnetic-activated cell sorting before cryopreservation preserves mitochondrial integrity in human spermatozoa.. Cell Tissue Bank.

[35] Lee TH, Liu CH, Shih YT, Tsao HM, Huang CC (2010). Magnetic-activated cell sorting for sperm preparation reduces spermatozoa with apoptotic markers and improves the acrosome reaction in couples with unexplained infertility.. Hum Reprod.

[36] Said T, Agarwal A, Grunewald S, Rasch M, Baumann T (2006). Selection of nonapoptotic spermatozoa as a new tool for enhancing assisted reproduction outcomes: an in vitro model.. Biol Reprod.

[37] Grunewald S, Reinhardt M, Blumenauer V, Said TM, Agarwal A (2009). Increased sperm chromatin decondensation in selected nonapoptotic spermatozoa of patients with male infertility.. Fertil Steril.

[38] Garner DL (2006). Flow cytometric sexing of mammalian sperm.. Theriogenology.

[39] Ramalho-Santos J, Varum S, Amaral S, Mota PC, Sousa AP (2009). Mitochondrial functionality in reproduction: from gonads and gametes to embryos and embryonic stem cells.. Hum Reprod Update.

[40] Amaral A, Ramalho-Santos J, St John JC (2007). The expression of polymerase gamma and mitochondrial transcription factor A and the regulation of mitochondrial DNA content in mature human sperm.. Hum Reprod.

[41] Amaral A, Ramalho-Santos J (2010). Assessment of mitochondrial potential: implications for the correct monitoring of human sperm function.. Int J Androl.

[42] Auger J, Leonce S, Jouannet P, Ronot X (1993). Flow cytometric sorting of living, highly motile human spermatozoa based on evaluation of their mitochondrial activity.. J Histochem Cytochem.

[43] Gallon F, Marchetti C, Jouy N, Marchetti P (2006). The functionality of mitochondria differentiates human spermatozoa with high and low fertilizing capability.. Fertil Steril.

[44] Romanato M, Cameo MS, Bertolesi G, Baldini C, Calvo JC (2003). Heparan sulphate: a putative decondensing agent for human spermatozoa in vivo.. Hum Reprod.

[45] Romanato M, Julianelli V, Zappi M, Calvo L, Calvo JC (2008). The presence of heparan sulfate in the mammalian oocyte provides a clue to human sperm nuclear decondensation in vivo.. Hum Reprod.

[46] Sousa AP, Tavares RS, Velez de la Calle JF, Figueiredo H, Almeida V (2009). Dual use of Diff-Quik-like stains for the simultaneous evaluation of human sperm morphology and chromatin status.. Hum Reprod.

[47] Yanagimachi R, Yanagimachi H, Rogers BJ (1976). The use of zona-free animal ova as a test-system for the assessment of the fertilizing capacity of human spermatozoa.. Biol Reprod.

[48] Rybouchkin A, Dozortsev D, de Sutter P, Qian C, Dhont M (1995). Intracytoplasmic injection of human spermatozoa into mouse oocytes: a useful model to investigate the oocyte-activating capacity and the karyotype of human spermatozoa.. Hum Reprod.

[49] Sutovsky P, Schatten G (2000). Paternal contributions to the mammalian zygote: fertilization after sperm-egg fusion.. Int Rev Cytol.

[50] Kasai T, Ogawa K, Mizuno K, Nagai S, Uchida Y (2002). Relationship between sperm mitochondrial membrane potential, sperm motility, and fertility potential.. Asian J Androl.

[51] Marchetti C, Jouy N, Leroy-Martin B, Defossez A, Formstecher P (2004). Comparison of four fluorochromes for the detection of the inner mitochondrial membrane potential in human spermatozoa and their correlation with sperm motility.. Hum Reprod.

[52] Sutovsky P, Moreno RD, Ramalho-Santos J, Dominko T, Simerly C (1999). Ubiquitin tag for sperm mitochondria.. Nature.

[53] WHO (2010). WHO Laboratory Manual for the Examination and processing of Human Semen. 5th Edition..

[54] Alvarez JG, Storey BT (1992). Evidence for increased lipid peroxidative damage and loss of superoxide dismutase activity as a mode of sublethal cryodamage to human sperm during cryopreservation.. J Androl.

[55] Ramalho-Santos J, Amaral A, Sousa AP, Rodrigues AS, Martins L, Mendez-Villas A, Diaz J (2007). Probing the structure and function of mammalian sperm using optical and fluorescence microscopy.. Modern Research and Educational Topics in Microscopy.

[56] Varum S, Bento C, Sousa AP, Gomes-Santos CS, Henriques P (2007). Characterization of human sperm populations using conventional parameters, surface ubiquitination, and apoptotic markers.. Fertil Steril.

[57] Palermo G, Joris H, Devroey P, Van Steirteghem AC (1992). Pregnancies after intracytoplasmic injection of single spermatozoon into an oocyte.. Lancet.

[58] Ramalho-Santos J, Amaral A, Brito R, Freitas M, Almeida Santos T (2004). Simultaneous analysis of cytoskeletal patterns and chromosome positioning in human fertilization failures.. Fertil Steril.

[59] Perreault SD, Barbee RR, Slott VL (1988). Importance of glutathione in the acquisition and maintenance of sperm nuclear decondensing activity in maturing hamster oocytes.. Dev Biol.

